# Architecturally robust design of ethylenediamine-assisted polyaniline/MXene nanohybrids for symmetric pouch-cell supercapacitors

**DOI:** 10.1039/d5na00692a

**Published:** 2025-10-09

**Authors:** Sithara Radhakrishnan, Subhashree Mohapatra, P. S. Anagha, Akshaya Shibu, K. Namsheer, Manasi Pathak, Sang Mun Jeong, Chandra Sekhar Rout

**Affiliations:** a Centre for Nano and Material Sciences, Jain (Deemed-to-be University), Jain Global Campus Kanakapura Road Bangalore 562112 Karnataka India r.chandrasekhar@jainuniversity.ac.in; b Department of Chemical Engineering, Chungbuk National University Cheongju Chungbuk 28644 Republic of Korea

## Abstract

Two-dimensional multi-layered MXenes have garnered significant research attention as an effective electrode material for supercapacitor fabrication, attributed to its exceptional mechanical and electrochemical properties. Nonetheless, MXenes face challenges like restacking issues, relatively lower electrochemical performance, and reduced electrical conductivity due to residual oxygen functional groups. A viable strategy to address these challenges involves intercalation and surface modification with ethylene diamine (EDA) molecules. In this study, we employed a two-step approach, beginning with a hydrothermal method to synthesize EDA-MXene, followed by *in situ* polymerization with aniline to produce a PANI/EDA-MXene hybrid. The PANI nanostructures that grow *in situ* on the surface-modified EDA-MXene nanosheets help mitigate the volume changes typically observed in PANI during charging/discharging cycles and facilitate faster charge transport across the surface. The surface-modified MXene nanosheets with EDA molecules serve as a foundational framework for the homogeneous growth of PANI nanorods, which enhance electrode–electrolyte interactions. Dunn analysis revealed a significant capacitive contribution thereby improving the overall charge storage capacity. At an optimized concentration of EDA and PANI, the PANI(ii)/MXene-EDA(ii) composite was utilized for the fabrication of symmetric pouch-cell supercapacitors. The device achieved a maximum areal capacitance of 668 mF cm^−2^ at 0.5 mA cm^−2^, with 95% capacitance retention over 8000 consecutive charge–discharge cycles. Furthermore, it delivered a maximum energy density of 59.3 Wh cm^−2^ and a power density of 778.3 W cm^−2^, indicating its potential for real-time applications. The synergistic effects of the EDA-intercalated MXene sheets, which prevent restacking issues, and the pseudocapacitive behaviour of the conducting polymer resulted in enhanced charge storage capacity.

## Abstract 2

The effective integration of EDA-intercalated MXene with *in situ* polymerized PANI successfully overcomes typical MXene challenges, resulting in enhanced supercapacitor capacitive performance which could be beneficial for real-time usage.

## Graphical abstract

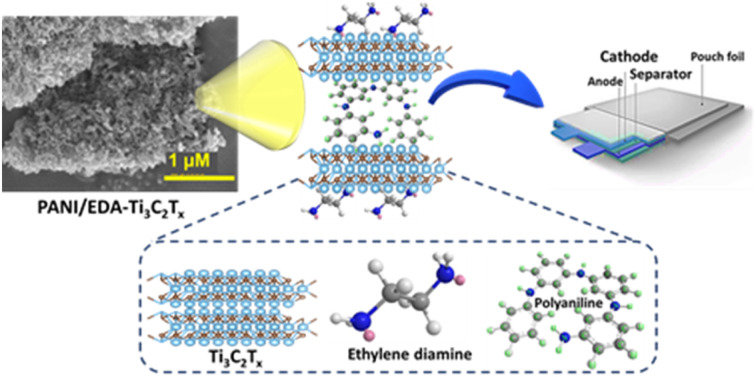

## 1. Introduction

The advancement of two-dimensional (2D) high-performance electrode materials is crucial for progress in the domains of energy conversion and storage.^1^ In this perspective, 2D MXenes have garnered significant interest as energy storage materials due to their beneficial properties, such as metallic conductivity, surface hydrophilicity, and the capacity to repeatedly intercalate and deintercalate ions.^2,3^ In contrast to other 2D materials, the presence of surface terminal functional groups and excellent metallic conductivity nature of MXenes favour faster redox reactions and increased charge storage capacity.^4–6^ With escalating demand for developing efficient energy storage devices, MXene based electrode materials stand as a promising contender for supercapacitor (SC) applications owing to their exceptional mix of high conductivity, large surface area, variable surface chemistry, and scalability.^7,8^ They exhibit pseudocapacitive behaviour where the charge storage is possible through the ion intercalation process where different ions intercalate the MXene layers reversibly and occupy electrochemically active sites on the MXene surface, contributing to the energy storage mechanism. This characteristic enhances the overall capacitance of SCs, leading to increased energy storage capacity.^7,9–12^ Due to their excellent electrochemical properties, they stand also as a promising alternative to limited capacity of carbon based materials which have attracted extensive research interest.^13,14^

Among the different reported MXene phases, Ti_3_C_2_T_*x*_ is commonly studied due to its superior intercalation pseudocapacitance behaviour, high metallic conductivity (6700 S cm^−1^) and electronic conductivity.^15–17^ However, the pristine MXene materials exhibit poor performance due to their restacking issues resulting in low electroactive surface area, and impeding the electrode–electrolyte interaction.^15,18–20^ To address this challenge, the functionalization of MXenes is one of the promising approaches to enhance the electrochemical performance where the MXene material surface is modified with various chemical functional groups or nanomaterials.^16,21^ For instance Zhu *et al.* prevented stacking of Ti_3_C_2_T_*x*_-MXene layers by using MgO particles as a hard template. After the template removal under acidic conditions, the assembled layers showed crumpled morphology with an enlarged interlayer space that delivered a capacitance of 180 F g^−1^ compared to the pristine MXene–based electrode that displayed 82 F g^−1^.^15^ Different kinds of inorganic cations such as Mg^2+^, Al^3+^, K^+^, Na^+^, and NH_4_^+^ have been electrochemically intercalated into the layers of Ti_3_C_2_ MXene, resulting in an improved electrochemical capacitance exceeding 300 F cm^−3^. However, research on 2D MXenes as supercapacitor electrode materials has been limited, particularly with the enlargement of interlayer space achieved through the intercalation of larger species, like organic molecules. A notable example is the recently reported ethylenediamine (EDA)-intercalated MXene. Xu *et al.* reported that an EDA modified MXene (EDA-Ti_3_C_2_T_*x*_) material, featuring expanded interlayer spacing was synthesized using a straightforward hydrothermal method. This process allowed for the effective production of a self-supporting film while avoiding oxidative degradation through simple vacuum filtering. In this structure, the EDA molecules were intercalated into the interlayer space of MXenes, forming N–Ti coordination bonds as the EDA molecules anchored themselves to the MXene nanosheets. This resulted in high gravimetric/volumetric specific capacitance properties (486.2 F g^−1^ at a scan rate of 2 mV s^−1^), high rate capability and good cycling stability (94.3% after 10 000 cycles) in the presence of H_2_SO_4_ as electrolyte.^22^ Given these outstanding characteristics, EDA-Ti_3_C_2_T_*x*_ is considered an excellent substrate for loading pseudocapacitive materials to further enhance its electrochemical performances.^23^ On the other hand, polyaniline (PANI), a member of the conducting polymer family, is viewed as a promising pseudocapacitive material that can be combined with carbon materials due to its superior flexibility, environmental stability and higher theoretical capacitance.^24^ They are mixed with 2D layered materials as it control the volumetric expansion during the charge–discharge process, thereby increasing the capacitive performance.^4,25^ It is being seen that synthesizing MXene/PANI-based composites for SCs could prevent restacking and agglomeration of MXene nanosheets effectively which ameliorates electrochemical properties.^24,26^ Wang *et al.* synthesized PANI nanoparticles as intercalators to regulate the Ti_3_C_2_T_*x*_ MXene nanosheet interlayer distance by the self-assembly method. The interlayered PANI nanoparticles not only restrain MXene self-stacking but also facilitate the ion-transportation process and build interconnected conductive channels. Benefitting from these intriguing properties, a symmetric SC was assembled involving a Ti_3_C_2_T_*x*_/PANI hybrid composite that showed an areal capacitance of 900 mF cm^−2^ and good cycling stability compared to pure MXene based symmetric SCs.^13^

In this scenario, we present an efficient and scalable method for synthesizing PANI/MXene-EDA nanohybrids. The organic molecule EDA was intercalated into the Ti_3_C_2_T_*x*_ nanosheets *via* hydrothermal methods, where EDA avoided the stacking issues by attaching to the Ti_3_C_2_T_*x*_ sheets through N–Ti coordination bonds. Then with the assistance of *in situ* polymerization, aniline monomers are able to attach to the EDA-intercalated MXene nanosheets due to the electronegative nitrogen atoms and residual functional groups such as oxygen on the MXene surface. This enabled the formation of PANI nanostructures on the substrate of the EDA-intercalated Ti_3_C_2_T_*x*_ layers that helped in showing high capacitive performance compared to the pristine Ti_3_C_2_T_*x*_, PANI, and Ti_3_C_2_T_*x*_-EDA based electrode materials. Furthermore, for real time usage, a symmetric pouch-cell SC was fabricated using the optimised PANI(ii)/MXene-EDA(ii) composite that showed an energy density (ED) of 59.3 Wh cm^−2^ and power density (PD) of 778.3 W cm^−2^ in 1 M H_2_SO_4_ electrolyte. Henceforth such rational designing of MXene-based hybrid composites involving the organic molecules and conducting polymers could solve the inherent problems of MXenes and further exploiting them toward developing advanced energy storage devices.

## 2. Experimental section

### 2.1 Synthesis of MXene (Ti_3_C_2_T_*x*_)

For the synthesis of Ti_3_C_2_T_*x*_, the MAX phase Ti_3_AlC_2_ was procured from Carbon, Ukraine Ltd with particle size ≤ 200 micron. The solution for etching Al was made by adding LiF (1 gm) to 20 mL of 9 M HCl and stirring continuously for 5 minutes. For the “bulk-etching” approach, 1 gm of Ti_3_AlC_2_ (MXene) powder was gradually added over 5 minutes to the LiF + HCl solution. The resulting solution was then stirred continuously for 48 hours at RT. Following this, the mixture was centrifuged in deionized (DI) water for 5 minutes at 3500 rpm. The supernatant was removed, and fresh DI water was introduced before the subsequent centrifugation cycle. This washing procedure continued until the pH of the supernatant became neutral.^27^

### 2.2 Synthesis of MXene -EDA

Ti_3_C_2_T_*x*_ (120 mg) was dispersed in distilled water (60 ml) and EDA of varying concentration was added (24 ml (MXene-EDA(i)), 48 ml MXene-EDA(ii) and 72 ml MXene-EDA(iii)) solution drop by drop and ultrasonicated for 30 minutes. The solution thus obtained was placed in a 100 ml Teflon-lined autoclave, sealed, and held in a hot air oven at 120 °C for 24 hours. Afterward, it was allowed to cool to room temperature naturally. The resulting product was collected and washed with distilled water until the pH was neutral. An additional wash with ethanol was performed to eliminate impurities and unreacted monomers. Finally, the sample was dried in a vacuum oven at 60 °C for another 24 hours, resulting in the final product.

### 2.3 Preparation of PANI/MXene -EDA

5 ml of aniline was added dropwise to 50 ml of a 1 M HCl solution in a beaker, which was then placed in an ice bath to cool to −4 °C while being stirred vigorously. MXene-EDA samples (30 mg for P(i)-MXene-EDA(ii), 50 mg of P(ii)-MXene-EDA(ii), and 70 mg of P(iii)-MXene-EDA(ii)) were dispersed in 10 ml of 1 M HCl. The homogeneous solution obtained was added dropwise to the aniline solution. Next, a solution of ammonium persulfate (APS) was prepared by dissolving 0.65 g of APS in 40 ml of 1 M HCl. This APS solution was then added dropwise to the aniline solution containing MXene-EDA while maintaining a temperature of −4 °C under stirring. The mixture was then stirred on a magnetic stirrer for 8 hours. The dark green product thus obtained was washed first with 1 M HCl, and then rinsed with DI water until the pH was neutral. An ethanol wash was performed to remove impurities and unreacted monomers. Finally, the sample was dried for 24 hours in a vacuum oven at 60 °C to get the final product.

## 3. Results and discussion

### 3.1 Structural, morphological, and elemental composition analysis

**Fig. 1:**
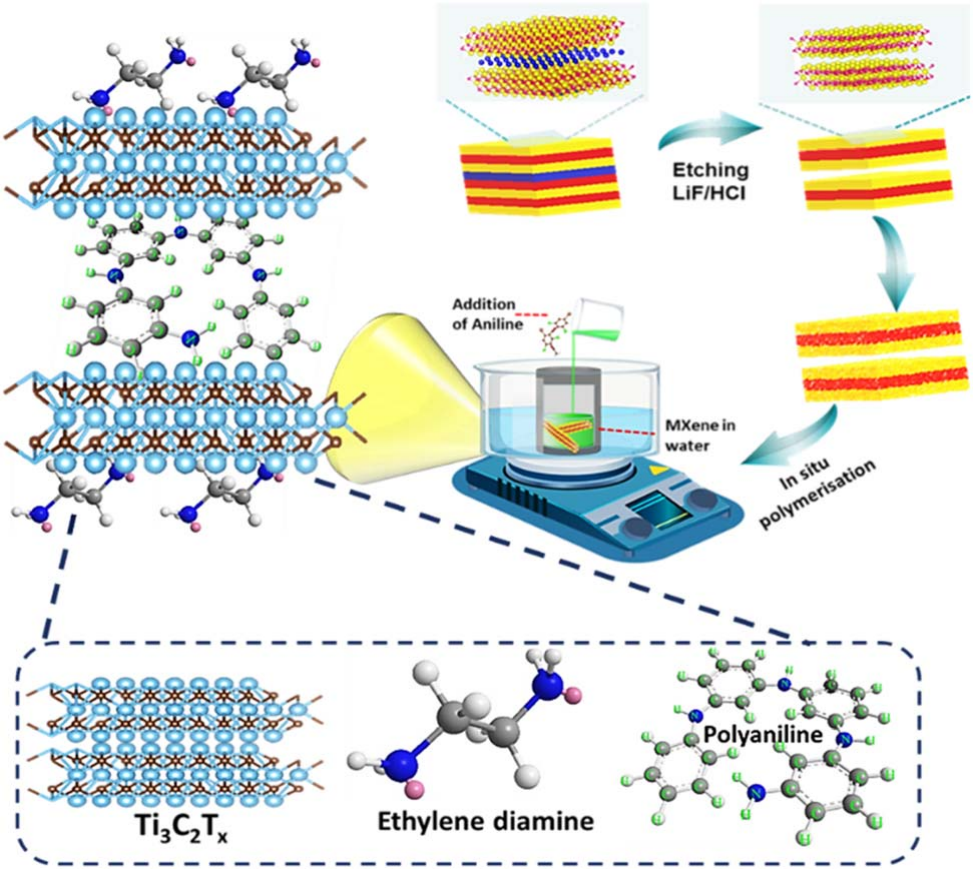
Schematic representation of the PANI/MXene-EDA.

The synthesis procedure begins with the preparation of a 2D accordion-like MXene by selectively etching the aluminum layer using LiF/HCl as the etchant. Following this, EDA molecules are intercalated into the layers of MXenes in a hydrothermal environment. Finally, the EDA-intercalated MXene undergoes *in situ* polymerization with aniline, resulting in the development of the PANI/MXene-EDA hybrid. Fig. 1 illustrates the synthesis procedure for PANI/MXene-EDA.

**Fig. 2:**
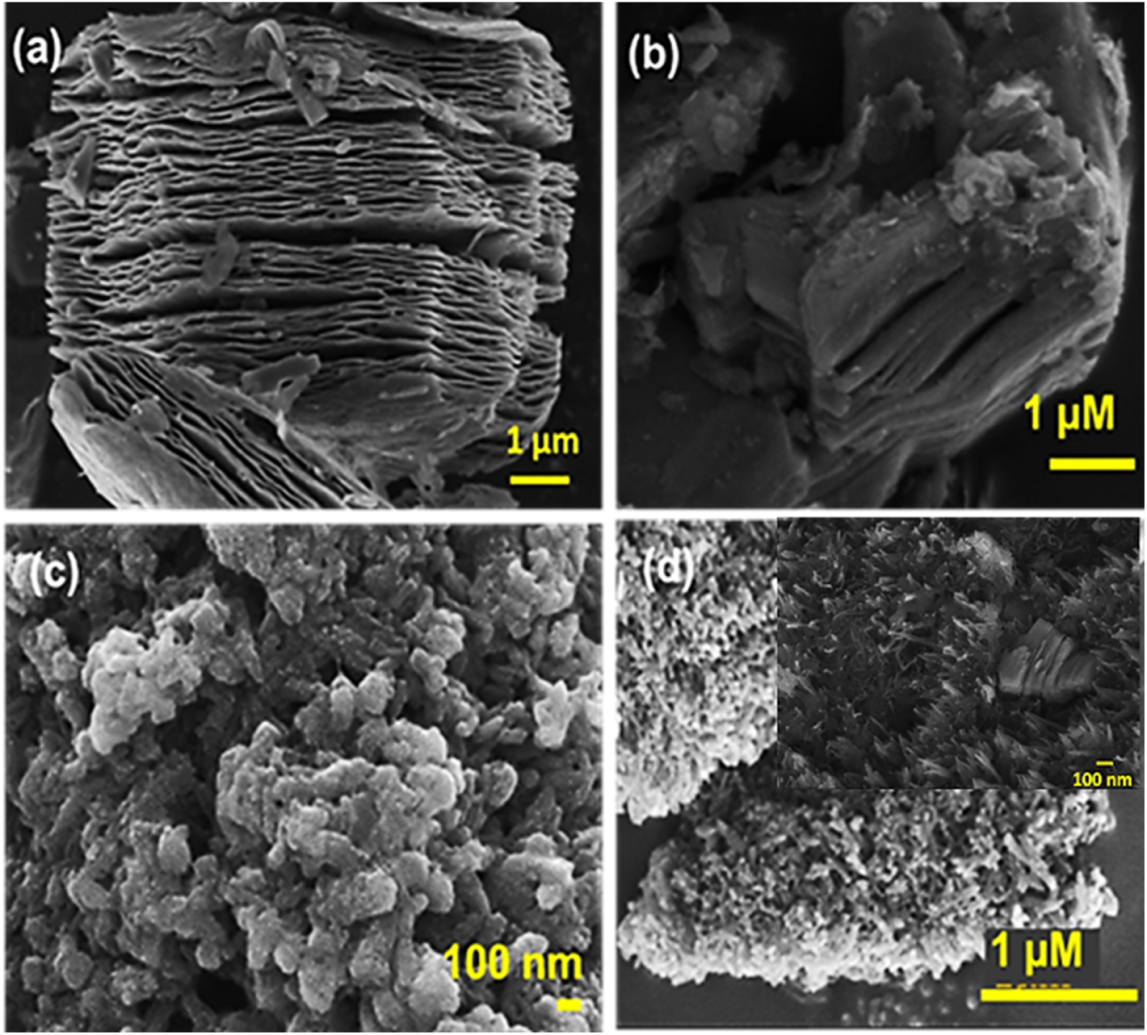
FESEM images of (a) MXenes, (b) EDA intercalated MXene, (c and d) PANI and PANI/MXene-EDA, respectively.

For morphological analysis, the FESEM images of MXenes, EDA-intercalated MXene, PANI and PANI/MXene-EDA materials are shown in Fig. 2. This characteristic accordion-like multi-layered Ti_3_C_2_T_*x*_ MXene sheet like morphology (Fig. 2(a)) is a direct outcome of the etching of Ti_3_AlC_2_ using LiF/HCl. The MXene-EDA sample's nanosheets remained intact, as illustrated in Fig. 2(b); however minor wrinkles were visible after the intercalation process. In contrast, the pure PANI shown in Fig. 2(c) has a rod-like shape with irregularly formed, agglomerated connected clusters. Then *in situ* polymerisation of PANI onto MXene-EDA composites produces a homogeneous network of PANI nanorods as seen in Fig. 2(d). During the first stage of polymerisation, aniline monomers adsorb onto the MXene-EDA nanosheet surfaces. Once polymerisation commences with the addition of APS, MXene-EDA nanosheets serve as a supporting framework for the formation of PANI nanorods. As the reaction advances, the initially formed nanorods serve as sites for the nucleation and for further PANI growth, ultimately leading to an extended, interconnected chain-like network that alters the surface characteristics of the sheets. Notably, this extensive interconnected network in the PANI/MXene-EDA hybrid enhances the material's porosity, which increases the electrochemically active surface area available to electrolyte ions, ultimately improving the SC performance.

**Fig. 3:**
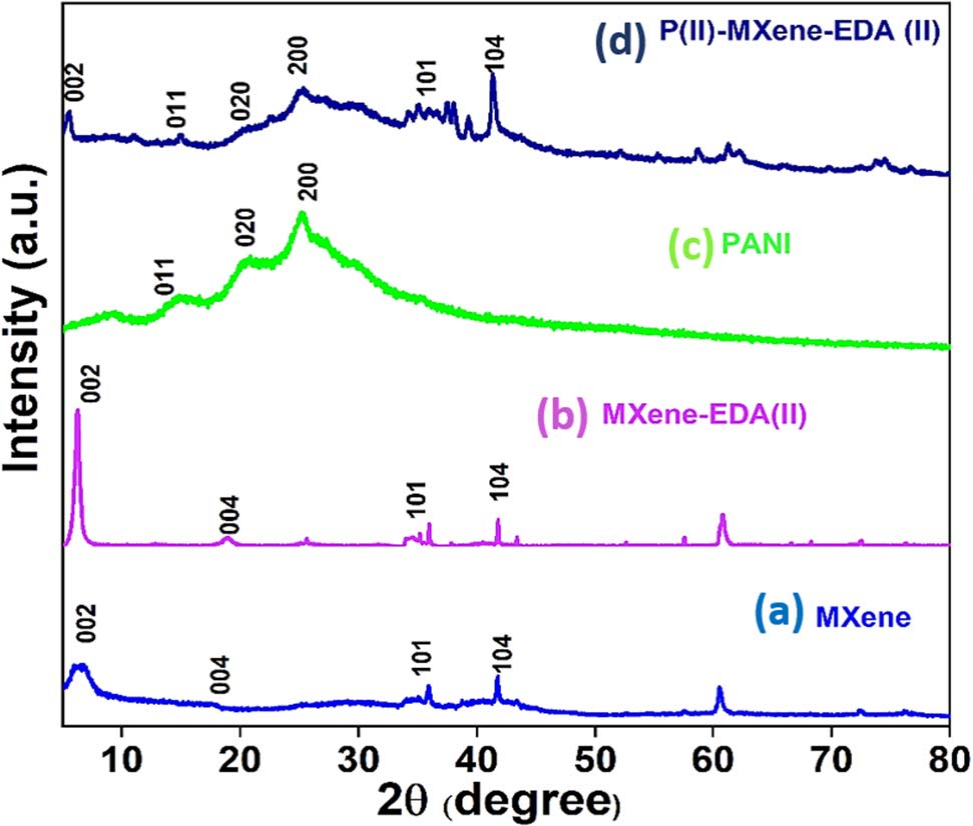
XRD spectra of (a) MXenes, (b) EDA intercalated MXene, (c) PANI and (d) PANI/MXene-EDA.

The crystallographic features of the synthesized samples were identified from the XRD pattern. The XRD spectra of MXenes, EDA-MXene, PANI, and PANI/MXene-EDA are shown in Fig. 3. Following the etching process the sharp peaks at 9.42° (002), 19.1° (004), 33.9°(101), 38.7°(104), 41.7° (105) and 48.5° (107) corresponding to Ti_3_AlC_2_ disappeared and a broad peak appeared at 6.72° (002). But for EDA-MXene this peak shifted to 6.46° and displays an increased intensity. This indicates an improvement in the crystallinity of the EDA-intercalated MXene nanosheets as well as an increase in the interlayer spacing due to insertion of EDA molecules.^22^ PANI has three distinct but less intense diffraction peaks at 14°, 20°, and 24°, which correspond to the (011), (020), and (200) crystal planes, respectively. The peaks at 14° and 24° represent the periodicity along and perpendicular to the polymer chain, respectively. The diffraction pattern of PANI/MXene-EDA is comparable to that of PANI, indicating that the well-dispersed MXene sheets act as a substrate for polymer chain growth. Notably, the (002) peak of MXenes stays intact after polymerisation with PANI, showing that the PANI/MXene-EDA hybrid structure was successfully synthesized.

**Fig. 4:**
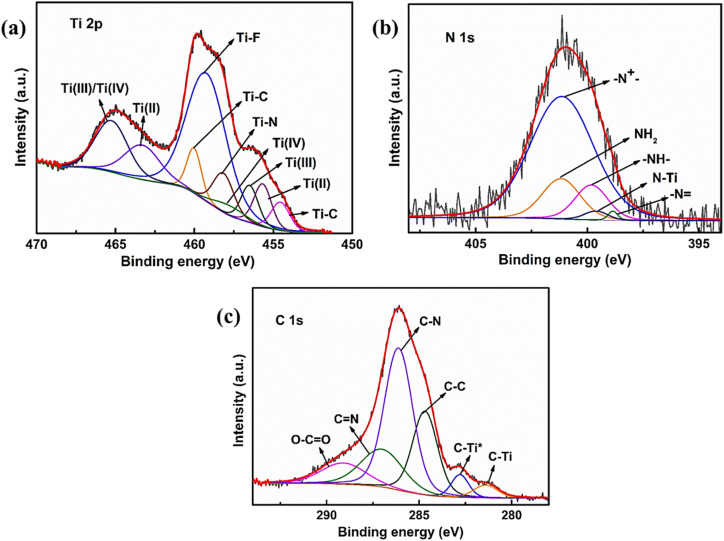
High resolution XPS spectra of (a) Ti 2p, (b) N 1s, and (c) C 1s.

The elemental compositions and their oxidation states in the hybrid PANI/MXene-EDA composite were investigated by XPS analysis. The deconvolution of Ti 2p spectra as seen in Fig. 4(a) revealed Ti 2p_1/2_ and Ti 2p_3/2_ in an area ratio of 2 : 1 where peaks at 455.1 eV and 461.2 eV were due to the Ti–C bond; 455.8 eV and 462.4 eV were assigned to Ti(ii); 457 eV and 464.2 eV were attributed to Ti(iii); 457.9 eV and 464.2 eV were attributed to Ti(iv); and 459.3 eV was attributed to the Ti–F bond. A further peak at 458.4 eV was ascribed to the Ti–N bond which confirmed the successful intercalation of EDA molecules into the Ti_3_C_2_T_*x*_ nanosheets. The polarity of Ti–N bonds enables the adsorption of electrolyte ions on the surface of the electrode and improves the overall structural stability of the hybrid composite.^22^ The high-resolution N 1s spectrum (Fig. 4(b)) showed peaks centered at 398.8 eV, 399.7 eV, and 401.2 eV that were ascribed to nitrogen functional groups like quinoid imine (–N=), benzenoid amine (–NH–), and cationic nitrogen (–N^+^–) groups, respectively. This confirmed the presence of PANI in the composite. Further peaks at 399.1 and 401.0 eV showed the existence of a N–Ti coordination bond due to reaction of NH_2_ with surface functional groups and the NH_2_ group, respectively. Fig. 4(c) displays the high-resolution C 1s spectrum which exhibits peaks at 282.1 eV, 282.7 eV, 284.6 eV, 285.6 eV, 286.9 eV and 288.9 eV ascribed to C–Ti, C–Ti*, C–C, C–N, C=N, and O–C=O bonds, respectively.^22,28^

### 3.2 Electrochemical performance evaluation

**Fig. 5:**
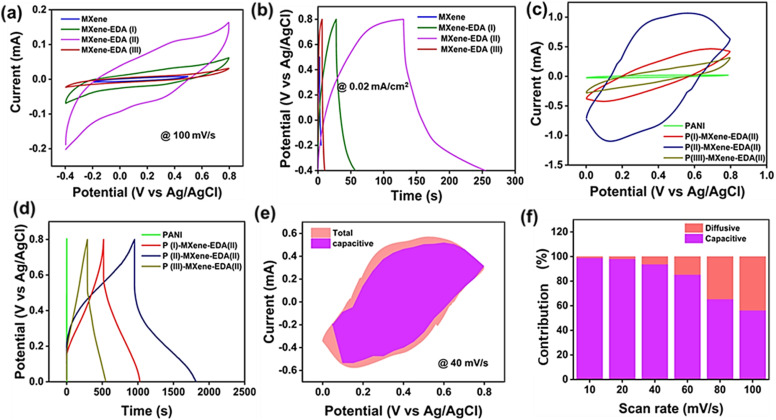
(a) CV curves at 100 mVs^−1^, (b) GCD curves at 0.02 mA cm^−2^ of pristine MXenes and MXene-EDA variants, (c and d) CV and GCD curves of pristine PANI, P(i)-MXene-EDA(ii), P(ii)-MXene-EDA(ii), and P(iii)-MXene-EDA(ii), (e) CV curves of P(ii)-MXene-EDA(ii) deconvoluted into capacitive and diffusive contribution, and (f) normalized capacitive and diffusive contribution at different scan rates.

First, the electrochemical performance of EDA-MXene electrodes after successful intercalation of EDA molecules was studied using a three-electrode configuration in 1 M H_2_SO_4_ electrolyte. When comparing the CV curves of MXenes with those of MXene-EDA, the latter exhibited a distinct quasi-rectangular shape in 1 M H_2_SO_4_ as seen from Fig. 5(a). This behaviour reflects the typical nature of faradaic pseudocapacitance, arising due to the protonation of oxygen groups on the surfaces and variation in the oxidation state of titanium atoms within the MXene nanosheets. Furthermore, the MXene-EDA composite performed noticeably better when the EDA concentration was increased from 24 mL to 48 mL. More specifically, the larger area of the CV curves for MXene-EDA(ii), indicating a greater charge storage capacity, suggests that the energy storage capacities were improved. This enhancement is mostly caused by the fact that when EDA molecules intercalate within the MXene structure, more electroactive sites become available. Better charge transfer during electrochemical processes was also made possible by the hybrids resulting in improved electrochemical activity. The increase in EDA concentration most likely resulted in increased surface area and porosity, allowing for enhanced interaction between electrolyte ions and the active material. Furthermore, the increased presence of functional groups from EDA promotes faradaic reactions, which are required for pseudocapacitance. However, at a concentration of 72 mL MXene-EDA(iii), performance is declining. The excess concentration of EDA can result in greater insulation, lowering overall conductivity and negatively impacting the composite's specific capacitance.^29^ As a result, modest increases in EDA concentration can improve performance, whereas excessive quantities may impair the intended electrochemical properties. Fig. 5(b) shows the GCD curves of the MXene and MXene-EDA electrodes. The enhanced performance of MXene-EDA(ii) is highlighted by its GCD profile, which deviates from a triangular shape and features plateau-like characteristics. This indicates a significant role of faradaic reactions, consistent with pseudocapacitive charge storage behavior. These characteristics imply that the electrodes have high coulombic efficiency and can support an efficient charge/discharge process. Fig. 5(c) presents the CV curves of PANI, which exhibit two distinct pairs of redox peaks, indicating that the energy storage capability is primarily due to the electrode’s pseudocapacitance behavior. Additionally, the effects of varying the concentration of PANI from 30 mL to 70 mL on MXenes were explored. Notably, the PANI(ii)/MXene-EDA(ii) hybrid shows a larger enclosed CV curve and high redox current, suggesting enhanced charge storage capabilities compared to pristine MXene and PANI. This is further corroborated from the GCD plot as visualized in Fig. 5(d), where the PANI(ii)/MXene-EDA(ii) composite demonstrates a longer discharge time relative to PANI(i)/MXene-EDA(ii), PANI(iii)/MXene-EDA (ii), and pristine PANI thus indicating superior charge storage capacity. All composite electrodes, including PANI, exhibit deviations from the triangular GCD shape, confirming their pseudocapacitive nature. The increased performance of PANI(ii)/MXene-EDA(ii) could be attributed to larger surface area and an extensive interconnected crosslinking network, as highlighted from the FESEM results. The presence of chain-like PANI nanostructures on the EDA-intercalated MXene sheets provides additional electroactive sites across the entire electrode surface, thereby enhancing rate performance. Additionally, the synergistic properties of both PANI and EDA-intercalated MXene lead to better performance of the hybrid, with the primary pseudocapacitance resulting from the reversible electrochemical reactions taking place on the surface of the PANI/MXene-EDA composite.

### 3.3 Charge storage mechanism of PANI/MXene-EDA

For elucidation of the charge-storage mechanism in the PANI(ii)/MXene-EDA(ii) hybrid, the Dunn technique was utilized to explore the kinetic mechanisms behind charge storage. This method allowed for a detailed deconvolution of all CV curves obtained from the PANI(ii)/MXene EDA(ii) sample into their capacitive and diffusive components. The results of this analysis indicate that capacitive-dominated electrochemical reactions are predominant across all scan rates, as shown in Fig. 5(e). Importantly, with an increase in scan rate the capacitive contribution decreases from 98% to 56% as depicted in Fig. 5(f). These revealed a significant capacitive contribution compared to the diffusion controlled reaction, highlighting distinct pseudocapacitive and electric double-layer involvement that together enhance the overall capacitive behaviour. This underscores the complex electrochemical processes occurring within the hybrid, involving reactions at multiple interfaces.

### 3.4 Pouch cell supercapacitors based on PANI/MXene-EDA

**Fig. 6:**
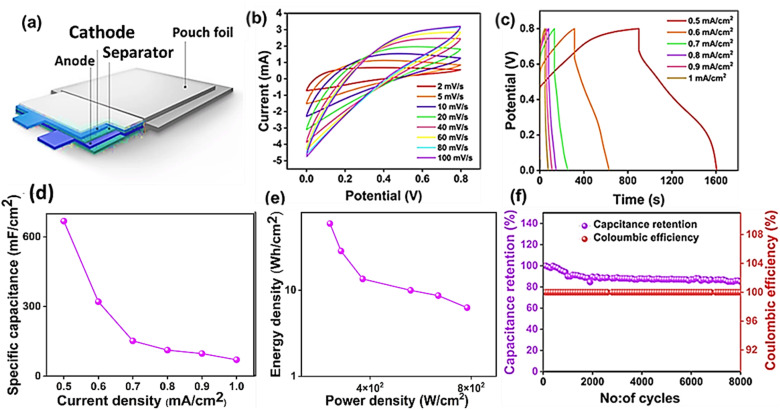
(a) Schematic demonstration of a pouch cell based on PANI(ii)/MXene-EDA(ii), (b) CV curves at different scan rates, (c) GCD curves at various current densities, (d) specific capacitance at different current densities, (e) Ragone plot, and (f) cycling stability of the symmetric pouch cell.

To investigate the practical uses of the optimised PANI(ii)/MXene-EDA(ii) composite, a symmetric pouch SC device was built with this optimized hybrid as both the positive and negative electrodes, which were coated on a steel mesh. Fig. 6(a) shows the schematic illustration of the fabricated symmetric pouch-cell device constituting a cathode, a anode and a separator followed by dipping in 1 M H_2_SO_4_ electrolyte and encapsulation of the device inside a pouch foil. The device operating voltage range was identified as 0 to 0.8 V, since individual electrodes in a three-electrode configuration give around 0.8 V, and thus the symmetric electrodes in the pouch-cell design are likewise predicted to deliver this voltage. The electrochemical stability of the produced symmetric pouch-cell SCs with PANI(ii)/MXene-EDA(ii) electrodes was evaluated by recording the CV curves of the SCs at different scan rates ranging from 10–100 mV s^−1^ as displayed in Fig. 6(b). The obtained current increased with the scan rate, indicating the durability of the electrode materials as well as the synergistic effects of the PANI and EDA-intercalated MXene sheets. The pouch-cell SCs showed excellent rate performance and reaction kinetics, as seen by the CV curves' near-symmetrical shape and lack of distortion.^30,31^ The GCD in a current range of 0.5 mA cm^−2^ to 1 mA cm^−2^ showed a linear profile, emphasising the pouch-cell device's superior electrochemical properties. A minimal IR drop of 0.1 V was recorded which signified the effective charge transport and the minimized internal resistance at the electrode/electrolyte interface. From Fig. 6(d), the pouch cell's areal capacitance (*C*_A_) at 0.5 mA cm^−2^ was found to be 668 mF cm^−2^ using eqn (S1). With the increase in current density, the *C*_A_ dropped to 70.6 mF cm^−3^ at 1 mA cm^−2^. Based on the Ragone plot shown in Fig. 6(e), the ED and PD of the device were estimated using eqn (S2) and (S3), resulting in a maximum ED of 59.3 Wh cm^−2^ and a PD of 778.3 W cm^−2^ at 0.5 mA cm^−2^. Lastly Fig. 6(f) shows the stability performance of the device for practical application that achieved 95% capacitance retention and 97% coulombic efficiency after 8000 successive cycles of charge–discharge. Table 1 highlights the potential of the PANI(ii)/MXene-EDA(ii) based symmetric pouch-cell device for energy storage purposes compared to the other reported work.

**Table 1:** Comparative performance of MXene based hybrid materials for symmetric supercapacitor devices

Material	Electrolyte	Capacitance	Energy density	Power density	Reference
PANI/GN/BC	1 M H_2_SO_4_	4.16 F cm^−2^	0.12 mWh cm^−2^	0.1 mW cm^−2^	[32]
Polyaniline/CNT/ethylene vinyl acetate	1 M H_2_SO_4_	192.3 mF cm^−2^	26.7 mWh cm^−2^	100 mW cm^−2^	[33]
Ti_3_C_2_T_*x*_/CNF/PC	PVA/KOH	143 mF cm^−2^	2.4 μWh cm^−2^	17.5 μW cm^−2^	[34]
Ti_3_C_2_T_*x*_ 3D aerogels	PVA/H_2_SO_4_	230 mF cm^−2^	38.5 μWh cm^−2^	1375 μW cm^−2^	[35]
Ti_3_C_2_/Copper/Cobalt hybrids	1 M H_2_SO_4_	290.5 mF cm^−2^	103.3 mWh cm^−2^	0.8 mW cm^−2^	[36]
Ti_3_C_2_/PANI	1 M H_2_SO_4_	900 mF cm^−2^	90.3 μWh cm^−2^	380 μW cm^−2^	[13]
**PANI/MXene-EDA**	**0.5 M H**_**2**_ **SO**_**4**_	**668 mF cm^−^** ^**2**^	**59.3 Wh cm** ^**−2**^	**778.3 W cm^−^** ^**2**^	**Our work**

## 4. Conclusion

In summary, a straightforward two-step synthetic approach was utilized, beginning with the hydrothermal intercalation and surface modification of MXene nanosheets using ethylene diamine (EDA), followed by the *in situ* polymerization of aniline to produce a robust PANI/EDA-MXene hybrid composite. The strategic surface modification with EDA molecules provided a crucial foundational framework, effectively mitigating the inherent restacking of MXene nanosheets. The subsequent *in situ* growth of PANI nanostructures on these EDA-modified surfaces not only enhanced electrode–electrolyte interactions but also significantly alleviated the characteristic volume expansion/contraction observed in PANI during charge/discharge cycles, while simultaneously facilitating more rapid charge transport kinetics across the composite structure. When the optimized PANI(ii)/MXene-EDA(ii) composite was integrated into a symmetric pouch-cell supercapacitor configuration, it exhibited outstanding electrochemical performance. The fabricated device achieved a remarkable maximum *C*_A_ of 668 mF cm^−2^ at a current density of 0.5 mA cm^−2^. Furthermore, it demonstrated exceptional long-term stability, maintaining 95% capacitance retention over 8000 consecutive charge–discharge cycles. The improved performance can be attributed to the large surface area and extensive interconnected network of PANI nanostructures within the hybrid material. This research unequivocally demonstrates a viable and effective strategy for designing high-performance, stable supercapacitor electrodes, highlighting the immense potential of such multifunctional hybrid composites for integration into advanced, real-time energy storage applications.

## Author contributions

Sithara Radhakrishnan: writing – original draft, investigation; Subhashree Mohapatra: writing – review and editing, data curation; Anagha P. S.: methodology, investigation; Akshaya Shibu: methodology, investigation; Namsheer K.: formal analysis, validation; Manasi Pathak: formal analysis, validation; Sang Mun Jeong: supervision, funding acquisition; Chandra Sekhar Rout: supervision, funding acquisition.

## Conflicts of interest

The authors have no relevant financial or non-financial interests to disclose.

## Supplementary Material

NA-007-D5NA00692A-s001

## Data Availability

The corresponding author can provide the data supporting the study's conclusions upon reasonable request. Supplementary information: material characterization; electrochemical characterization; symmetric pouch cell fabrication. See DOI: https://doi.org/10.1039/d5na00692a.
