# Supplementary material for: Architecturally robust design of ethylenediamine-assisted polyaniline/MXene nanohybrids for symmetric pouch-cell supercapacitors
Source: Nanoscale Adv. 2025 Oct 9;7(24):7950–7. doi: 10.1039/d5na00692a (PMC12550595; doi:10.1039/d5na00692a)
Supplement: NA-007-D5NA00692A-s001 [file NA-007-D5NA00692A-s001.pdf]

Supplementary Information (SI) for Nanoscale Advances.
This journal is © The Royal Society of Chemistry 2025

Electronic Supplementary Information

Architecturally Robust Design of Ethylenediamine-Assisted Polyaniline/MXene Nanohybrids for Symmetric Pouch-Cell Supercapacitors

Sithara Radhakrishnan[a], Subhashree Mohapatra[a], Anagha PS[a], Akshaya Shibu[a], Namsheer KK[a], Manasi Pathak[a], Sang Mun Jeong[b], Chandra Sekhar Rout[*ab]

[a]*Centre for Nano and Material Sciences, Jain (Deemed-to-be University), Jain Global Campus, Kanakapura Road, Bangalore 562112, Karnataka, India.*

[b]*Department of Chemical Engineering, Chungbuk National University, Cheongju, Chungbuk 28644, Republic of Korea*

**Corresponding author:* r.chandrasekhar@jainuniversity.ac.in

S1. Material characterization

The phase and structural characterisation of samples was carried out using Rigaku Ultima IV having a NI-filter for Cu–Kα radiation, λ = 0.1541 nm at a scanning rate of 3 ^{o}/min. The morphology of prepared samples was determined with the help of field emission scanning electron microscopy (FESEM, JEOL JSM-7100F, JEOL Ltd., Singapore). The valence state and chemical compositions of the prepared samples were determined using Thermo Fisher ESCALAB Xi^{+}.

S2. Electrochemical characterization

The electrochemical characterisations were carried out on a CS Corrtest CS350 electrochemical workstation Version 5.3 from Wuhan using CS Studio software. The working electrode was constructed by dispersing 2 mg active material in nafion and IPA (1:19) solution, then drop-casted onto a glassy carbon electrode and dried at RT. The electrochemical performance of the prepared samples was investigated in three-electrode configuration constituting working electrode, saturated Ag/AgCl reference electrode, and Pt wire counter electrode in the presence of 1 M H_2SO_4 aqueous electrolyte. Electrochemical examinations included: (i) cyclic voltammetry (CV) studies at scan rate in the range 10-100 mV s^{-1}, (ii) galvanostatic charging/discharging (GCD) tests at different current density, and (iii) electrochemical impedance spectroscopy (EIS) analysis using an open-circuit voltage with an amplitude of 5 mV, spanning the frequency range of 100 kHz to 0.01 Hz.

S3. Pouch cell fabrication

For pouch cell fabrication, PANI/MXene-EDA, carbon black, and PVDF were blended in an 80:10:10 weight ratio and dry-ground using a mortar and pestle for 2 hours. NMP solvent was then added gradually to the mixture, followed by an additional 1 hour of grinding to obtain a homogeneous slurry. This slurry was then drop-casted onto stainless steel (SS) foil current collectors with a defined surface area of 1 cm × 1 cm. Each electrode contained 3 mg of active material. The electrodes were dried in a hot air vacuum oven at 70 °C for 12 hours. Finally, a symmetric cell was assembled using the two prepared electrodes, with 1 M H_2SO_4 serving as the electrolyte for electrochemical testing. The areal capacitance (C_A), energy density (ED), and power density (PD) were estimated from the charge–discharge profile curves using the following equations: [1]

$$C_A = \left(\frac{I * \Delta t}{A * \Delta V}\right) \qquad \text{(S1)}$$

Where, C_A is the specific capacitance (mF/cm^2), I is the current response (mA) of the electrode for unit area contact with electrolyte, Δt is the discharge time, and ΔV is the potential operation window.

$$ED = (\frac{1}{2} * C_A * (V_2 - V_1)^2)/3.6 \qquad \text{(S2)}$$

$$PD = (E/\Delta t)*3600 \qquad \text{(S3)}$$

Where C_A is the specific capacitance ($V_2 - V_1$) is the potential window (V_2 and V_1 are the final and initial potential values, respectively) and Δt is the discharge time.

References

1. S. Radhakrishnan, M. Monisha, S. R. Ka, M. Saxena, S. M. Jeong and C. S. Rout, Adv. Sustain. Syst., DOI:10.1002/adsu.202400529.
